# circRNF10 Regulates Tumorigenic Properties and Natural Killer Cell-Mediated Cytotoxicity against Breast Cancer through the miR-934/PTEN/PI3k-Akt Axis

**DOI:** 10.3390/cancers14235862

**Published:** 2022-11-28

**Authors:** Fei Liu, Yang Sang, Yang Zheng, Lina Gu, Lingjiao Meng, Ziyi Li, Yuyang Dong, Zishuan Wei, Cuizhi Geng, Meixiang Sang

**Affiliations:** 1 Research Center, The Fourth Hospital of Hebei Medical University, Shijiazhuang 050017, China; 2 Laboratory Animal Center, The Fourth Hospital of Hebei Medical University, Shijiazhuang 050017, China; 3 Breast Center, The Fourth Hospital of Hebei Medical University, Shijiazhuang 050017, China

**Keywords:** breast cancer, circRNF10, miR-934, PTEN, NK

## Abstract

**Simple Summary:**

circRNF10 is significantly downregulated in breast cancer (BC) tissues, and its downregulation is associated with a poor prognosis. circRNF10 regulates PTEN expression and the PI3k/Akt/MICA signaling pathway by sponging miR-934. Our findings revealed that circRNF10 functions as a novel anti-oncogene in BC, and overexpression of circRNF10 could enhance the killing efficiency of NK-92MI cells against BC cells.

**Abstract:**

Circular RNA (circRNA), a type of non-coding RNA, has received a great deal of attention with regard to the initiation and progression of tumors. However, the molecular mechanism and function of circRNAs in breast cancer (BC) remain unclear. In the current study, we discovered that hsa_circ_0028899 (also called circRNF10) was significantly reduced in BC tissues, and a higher level of circRNF10 was markedly related to a favorable prognosis. The results of CCK8, colony formation, Transwell, ELISA, and NK cell-mediated cytotoxicity assays indicated that increased circRNF10 expression could significantly repress the proliferation, invasion, and migration of BC cells and enhance the killing efficiency of NK cells against BC cells. According to these biological functions, the possible role and molecular mechanism of circRNF10 in BC cells were further investigated. We used bioinformatics prediction tools to predict circRNF10-bound miRNAs, which were verified by many experimental studies, including FISH, luciferase reporter assays, RIP, and Western blots. These data suggest that circRNF10 serves as a molecular sponge for miR-934 to further regulate PTEN expression and PI3k/Akt/MICA signaling in vitro and tumor growth in vivo. Altogether, these findings reveal that circRNF10 functions as a novel anti-oncogene in BC via sponging miR-934 and suppressing the PI3K/Akt/MICA pathway.

## 1. Introduction

Breast cancer (BC) is the most common cancer in women, accounting for the highest incidence of malignant tumors. According to the most recent epidemiological statistics, the number of BC patients in China is about 2.5 million. Based on the expression level of receptors, BC can be divided into four types: luminal A and B, Her-2 positive, and basal-like. Due to the heterogeneity of BC, some patients do not benefit from current conventional treatments [1]. Thus, it is essential to elucidate the molecular mechanism of BC progression and identify novel targets for prognosis and molecular therapy to improve the survival rate of BC patients.

Circular RNAs (circRNAs) are a new class of non-coding RNAs that form closed single-stranded circular RNA molecules through a procedure called back splicing [2,3]. circRNAs lack free 5′ caps and form 3′ tails, which makes them more stable than their linear counterparts [4]. Numerous researchers have found that circRNAs are aberrantly expressed in various carcinomas and have crucial functions in carcinoma biology [5,6,7]. The mechanism of action of circRNAs is diverse and complex. To date, accumulating studies have shown that circRNAs participate in the development of many kinds of tumors by serving as a scaffold for the assembly of protein complexes, regulating RNA splicing and gene transcription, translating into proteins or polypeptides, and acting as a microRNA sponge [8,9,10]. Among them, the latter is the most common mechanism of action of circRNA. Growing evidence has shown that many circRNAs are involved in tumorigenesis through competing endogenous RNA (ceRNA) mechanisms [11]. For example, the circular RNA ciRs7 can adsorb miR-7 to relieve the repression of its target genes [12,13]. circROCK1-E3/E4 suppresses tumor growth and metastasis by upregulating PTEN through miR-532-5p sponging [14]. Analogously, circ_001678 can sponge miR-326 to increase ZEB1 expression and promote immune escape in NSCLC [15].

Recently, growing evidence has shown that circRNAs play a major role in the initiation and progression of BC [16,17]. In this study, RNA-seq and circRNA data were obtained from the TCGA and GEO databases, respectively. The limma package was used to select differentially expressed circRNAs (DEcircRNAs). We discovered that hsa_circ_0028899 (termed circRNF10 in this study) was significantly downregulated in BC and indicated a poor prognosis for BC patients. Functionally, circRNF10 can suppress BC cell proliferation, invasion, and migration and enhance the killing efficiency of NK-92MI cells against BC cells. Mechanically, circRNF10 can act as a sponge for miR-934 to reverse the PI3k/Akt pathway by regulating PTEN expression, thereby suppressing the progression of BC. This work provides novel insights into the circRNA profile in BC and indicates that circRNF10 has the potential to serve as a novel target for molecular therapy of BC.

## 2. Materials and Methods

### 2.1. RNA-Sequencing and circRNA Microarray Data Collection and Bioinformatics Analysis

We downloaded the GSE101123 circRNA dataset from the Gene Expression Omnibus (GEO) database (http://www.ncbi.nlm.nih.gov/geo/0, accessed on 26 August 2019), which included eight BC tissues and three normal breast tissues. miRNA and mRNA expression profile data were obtained from The Cancer Genome Atlas (TCGA) database (https://portal.gdc.cancer.gov/repository, accessed on 3 September 2019); the miRNA data included 104 normal tissues and 1103 BC tissues, and the mRNA data contained 113 normal samples and 1109 BC samples.

We used |log2FC| ≥ 1 and P-adjust < 0.05 as thresholds to analyze the DEcircRNAs in the GSE101123 dataset. Using FDR < 0.05 and |log2FC| ≥ 1 as cut-off criteria, the differentially expressed miRNAs (DEmiRNAs) were screened with the Edge-R package. The differentially expressed mRNAs (DEmRNAs) were selected by using the Deseq package in R (FDR < 0.001 and |log2FC| ≥ 0.5).

### 2.2. Screening and Prediction of circRNA-Bound miRNAs and miRNA-Targeted mRNAs

We used the CSCD database (http://www.gb.whu.edu.cn/CSCD, accessed on 3 September 2019) to assess the binding sites of circRNAs and miRNAs. Then, we determined the intersection of predicted target miRNAs with DEmiRNAs by using a Venn diagram.

We used the TargetScan prediction tool (http://www.targetscan.org/vert_72/, accessed on 5 March 2022) to evaluate the miRNA-targeted mRNAs, and GeneCards (https://www.genecards.org/, accessed on 5 March 2022), to collect genes and targets related to BC (relevance score ≥ 100). Then we constructed a Venn diagram to determine the intersection of DEmRNAs and target genes predicted by TargetScan and GeneCards.

### 2.3. Tissue Specimens and Tissue Microarray

Thirty pairs of fresh frozen tumors and adjacent normal tissues from BC patients were obtained from the Breast Center of the Fourth Hospital of Hebei Medical University with the patients’ informed consent. These patients did not receive any treatment prior to surgery. In addition, 140 samples of breast cancer tissues derived from the HBreD140su06 tissue microarray were purchased from Shanghai Outdo Biotech. This research was approved by the Ethics Committee of the Fourth Hospital of Hebei Medical University.

### 2.4. Xenograft Tumor Model

Twenty 4-week-old female NOD/SCID mice were purchased from SPF Biotechnology Co., Ltd. (SiPeiFu, Beijing, China) and randomly divided into 4 groups. Each mouse was subcutaneously inoculated with vector-transfected MDA-MB-231 cells (5 × 10^6^). After tumor formation, the volume was measured every 6 days to calculate tumor growth. Four weeks later, the mice were euthanized, and the tumors were weighed. The Animal Care Committee of the Fourth Hospital of Hebei Medical University approved our animal experimentation procedures.

### 2.5. Cell Culture and Transfection

MDA-MB-231 and BT-549 cells were cultured in RPMI1640 medium (#8121581, Gibco, Carlsbad, CA, USA) supplemented with 10% FBS (#10099141, Gibco, Carlsbad, CA, USA) and penicillin/streptomycin in a humidified atmosphere of 5% CO_2_ at 37 °C. NK-92MI cells were cultured in ClonePlus^TM^ NK-92 MI cell line complete medium (#TCH-G293, Cas9X, Jiangsu, China). Overexpressing circRNF10 and the control vector were obtained from Geneseed Biotech (Guangzhou, China). miR-934 mimics and miR-NC were obtained from RiboBio (Guangzhou, China). For circRNA transfection, 3 μg plasmid was transfected with the FuGENE HD (#E2311, Promega, Madison, WI, USA). For miRNA transfection, BC cells were transfected with HiPerFect (#301704, Qiagen, Hilden, Germany) based on the manufacturer’s instructions.

### 2.6. DNA/RNA Extraction, RNase R Treatment and Actinomycin D Assay

Total RNA from MDA-MB-231 and BT-549 cells was isolated using TRIzol reagent (Invitrogen, Carlsbad, CA, USA). The genomic DNA (gDNA) from BC cells was extracted by a TIANGEN DNA extraction kit (#DP304, TIANGEN, Beijing, China). For the actinomycin D assay, BC cells were treated with actinomycin D prior to RNA extraction. For the RNase R assay, total cell RNA (2 μg) was digested with 3 U/μg RNase R reagent (#RNR07250, Biosearch Technologies, Petaluma, CA, USA) at 37 °C for 15 min and 85 °C for 3 min. Following treatment with RNase R and actinomycin D, circRNF10 expression and linear RNF10 were investigated using qRT-PCR.

### 2.7. Real-Time PCR Validation and Nucleic Acid Electrophoresis

Total RNA from cells and tissues was isolated using the TRIzol reagent (Invitrogen, Carlsbad, CA, USA). The corresponding cDNA was generated using GoScript^TM^ (#A5001, Promega, Madison, WI, USA). qRT-PCR was performed with SYBR Green Reagent (#A6002, Promega, Madison, WI, USA). The levels of circRNF10 and miR934 were measured using specific primers (RiboBio, Guangzhou, China). All primer sequences applied to PCR amplification are listed in Table S1. The RT-PCR amplification products were separated using 2% agarose gel and detected using UV irradiation.

### 2.8. Fluorescence In Situ Hybridization (FISH)

The CY3-labeled circRNF10 probe and the FITC-labeled miR-934 probe were purchased from Geneseed Biotech (Guangzhou, China). For FISH in BC cells, MDA-MB-231 and BT-549 cells were cultured on coverslips, fixed with 4% formaldehyde, and permeabilized in PBS with 0.5% Triton X-100. FISH probes were diluted, denatured, balanced, and added to breast cancer cells overnight at 37 °C. For FISH in tissues, the tissue microarray slides (Outdo Biotech, Shanghai, China) were dewaxed in xylene and ethanol solutions. The subsequent procedure was similar to that described for FISH in cells. After hybridization, the nuclei were stained with DAPI. Confocal laser scanning microscopy (Zeiss, Jena, Germany) was then performed. The probe sequences were as follows: circRNF10-CY3 5′CY3-tacaaatgcgctcctagatgaat-3′CY3; hsa-miR-934-FITC 5′FITC-ccagtgtctccagtagtaga- 3′FITC. According to the cytoplasmic expression intensity of circRNF10, samples were classified as follows: negative or faint expression in most cells was defined as the negative group; moderate expression in <50% of cells or low expression in most cells was defined as the low expression group; and moderate to strong expression in most cells was defined as the high expression group.

### 2.9. RNA Immunoprecipitation (RIP)

miR-934 mimics and a Myc-tagged AGO2 vector were co-transfected into the MDA-MB-231 cells. RIP assay was performed with the Magna RIP Kit (#17-700, Millipore, Billerica, MA, USA). MDA-MB-231 cells were lysed in RIP lysis buffer containing proteinase and RNase inhibitors. Magnetic beads were conjugated with anti-Myc (#ab9106, Abcam, Cambridge, UK) or anti-IgG (#ab172730, Abcam, Cambridge, UK) for 1 h. Then, cell lysates were immunoprecipitated using magnetic beads at 4 °C overnight. After purification, qRT-PCR was performed to obtain the abundance of targeted circRNAs.

### 2.10. Luciferase Promoter Assay

Wild-type circRNF10 with potential miR-934 binding sites and luciferase vectors were purchased from Geneseed Biotech Corporation (Guangzhou, China). Moreover, the wild-type (WT) and mutant (Mut) 3′UTR sequences of PTEN were cloned into psiCHECK2. These plasmids were cotransfected using FuGENE-HD transfection reagent in 6-well plates with miR-934 mimic or miR-NC into MDA-MB-231 cells. At 48 h after transfection, a dual-luciferase reporter assay system (#E1910, Promega, Madison, WI, USA) was performed to measure luciferase activity following the manufacturer’s instructions.

### 2.11. Transwell Migration and Invasion Assays

The cell migration and invasion abilities were investigated using Transwell chambers. For cell migration analysis, MDA-MB-231 and BT-549 cells (4 × 10^4^) were placed into the upper chambers (#3422, Corning Inc., Corning, NY, USA) with serum-free culture medium. For cell invasion assays, a 1:7 concentration of Matrigel (#354248, BD, Franklin, NJ, USA) was used in the upper chamber, and the lower chamber was filled with culture medium supplemented with 10% FBS. After 24 h, the upper chamber cells were wiped. The BC cells that invaded the bottom of the membrane were stained with crystal violet and counted using a light microscope (magnification, 200×) in 3 randomly selected fields.

### 2.12. Cell Proliferation Assay

The cell proliferation assay was measured by a Cell Counting Kit-8 (CCK-8). MDA-MB-231 and BT-549 cells (2 × 10^3^/well) were seeded in a 96-well plate, and CCK-8 (10 μL) was added to each well at the same time every day. After 1 h of incubation at 37 °C, the absorbance of the cell at 450 nm was read by a multifunctional microplate reader.

### 2.13. Colony Formation Assay

MDA-MB-231 and BT-549 cells transfected with corresponding plasmids were seeded into 6-well plates (2 × 10^3^/well). Then, cells were placed in a 37 °C sterile incubator for 12 days. Cell colonies were stained using a crystal violet solution. Colonies were then observed.

### 2.14. Lactate Dehydrogenase (LDH) Assay

The cytotoxicity of NK-92MI cells against BC cells was assessed by LDH activity using a CytoTox 96^®^ Non-Radioactive Cytotoxicity Assay Kit (#G1780, Promega, Madison, WI, USA). Briefly, breast cancer cells were transfected with pLC5-ciR, circRNF10, miR-934, miR-NC, pLC5-ciR+miR-NC, pLC5-ciR+miR-934, circRNF10+miR-NC, and circRNF10+miR-934. After 24 h, NK-92 MI cells were co-cultured with transfected MDA-MB-231 cells at ratios of 5:1, 10:1, and 20:1 (target cells: effector cells) for 6 h. Then, cell culture supernatants were gathered to assess LDH release.

### 2.15. Cytokine Assay

The protein expression of tumor necrosis factor-a (TNF-a) and interferon-γ (IFN-γ) in cell supernatants were quantified using IFN-γ human ELISA Kit (#RK00015, ABclonal, MA, USA) and TNF-α human ELISA Kit (#KE00154, Proteintech, Rosemont, IL, USA) following the respective manufacturer’s instructions.

### 2.16. Western Blot Analysis

MDA-MB-231 and BT-549 cells in each group were lysed with radio immunoprecipitation assay (RIPA) lysis buffer containing PMSF (1 mM). The protein lysates were denatured by high temperatures and separated using 10% SDS polyacrylamide gel electrophoresis. The separated protein gel was then transferred to PVDF membranes and blocked using 5% skim milk. Then, primary antibodies were added overnight at 4 °C: anti-PTEN (#22034-1-AP, 1:1000; Proteintech, Rosemont, IL, USA), anti-β-catenin (#ab32572, 1:1000; Abcam, Boston, MA, USA), anti-PI3K (110α) (#67121-1-Ig, 1:1000; Proteintech, Rosemont, IL, USA), anti-p-PI3K p55 (Tyr199) (#ab278545, 1:1000; Abcam, Boston, MA, USA), anti-p-Akt (Ser473) (#ab81283, 1:1000; Abcam, Boston, MA, USA), anti-Akt (#ab179463, 1:1000; Abcam, Boston, MA, USA), and anti-MICA (#65161-1-Ig, 1:1000; Proteintech, Rosemont, IL, USA). After that, secondary antibodies (#SA00001, 1:5000; Proteintech, Rosemont, IL, USA) were incubated for 1 h. The membranes were stained with an ECL kit (#PE0010, Solarbio, Beijing, China).

### 2.17. Statistical Analysis

The SPSS 24.0 statistical software and GraphPad Prism software were used to analyze the statistical significance of all the experiments. ANOVA or the Student’s *t*-test was used to analyze quantitative data, and *p* < 0.05 was regarded as statistically significant.

## 3. Results

### 3.1. circRNAs Expression Profiles in BC

**Figure 1:**
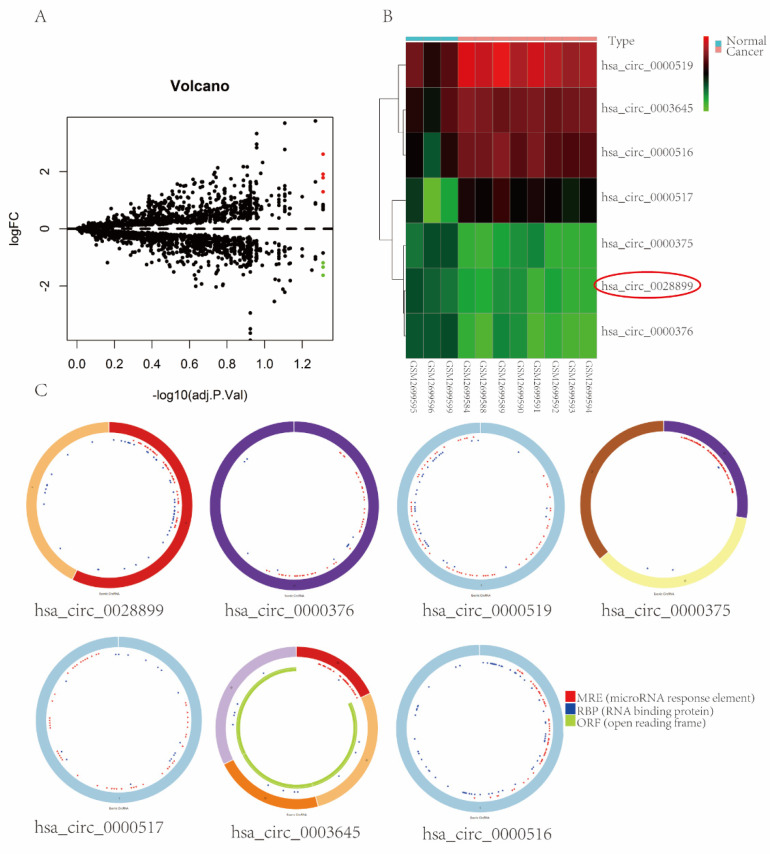
Expression profiles of seven DECs in GEO datasets and their structures. (**A**) Volcano plot of DECs. (**B**) Heatmap of DECs in BC. (**C**) Basic structure and characteristics of seven DECs predicted by the CSCD database.

We used |log2FC| ≥ 1 and P-adjust < 0.05 as thresholds to analyze the DEcircRNAs in BC tissues. We identified seven differentially expressed circRNAs (DECs). Among them, four DECs were upregulated, and three DECs were downregulated. Volcano plots (Figure 1A) and a heat map (Figure 1B) were used to display DECs. The basic structures and characteristics of the seven DECs are shown in Figure 1C and Table 1. In this study, we selected hsa_circ_0028899 (circRNF10 in this study) as our protagonist.

### 3.2. Characterization of circRNF10 in BC

**Figure 2:**
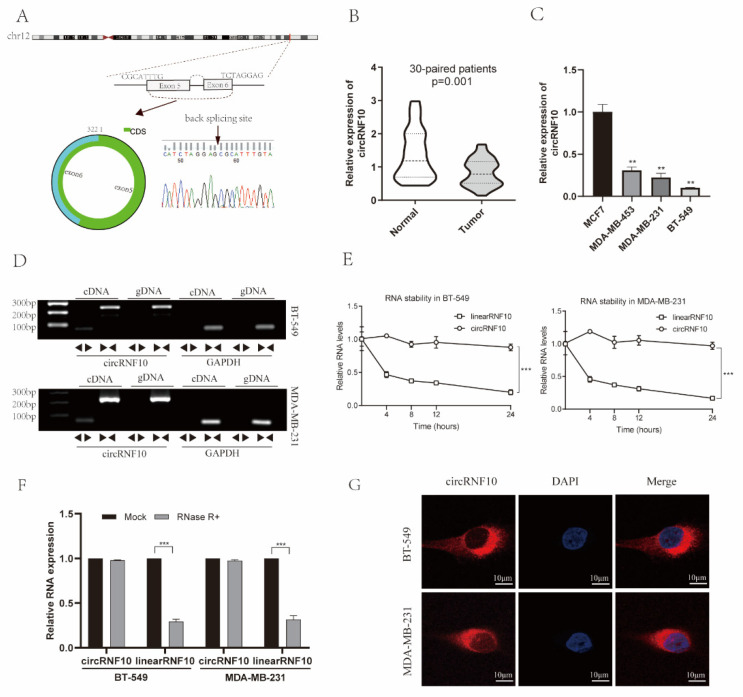
Characterization of circRNF10. (**A**) Schematic illustration of circRNF10 originating from exons five and six of RNF10 and Sanger sequencing of circRNF10 indicate a back-splice junction. (**B**) Violin plots for circRNF10 expression in BC by qRT-PCR. (**C**) Expression levels of circRNF10 in BC cells (MCF7, MDA-MB-453, MDA-MB-231, BT-549). Experiments were repeated thrice. ** *p* < 0.01. (**D**) Existence of circRNF10 was confirmed in BC cells by RT-PCR. Divergent primers were performed to amplify circRNF10 in cDNA rather than gDNA. (**E**) Relative RNA levels of circRNF10 and RNF10 mRNA under treatment with actinomycin D in BC cells. The experiments were repeated three times. *** *p* < 0.001. (**F**) mRNA expression of circRNF10 and RNF10 in RNase R-treated BC cells was analyzed by qRT-PCR. Experiments were repeated thrice. *** *p* < 0.001. (**G**) Subcellular localization of circRNF10 was determined by FISH. Scale bar, 10 μm.

circRNF10 was derived from exons five and six of the host gene RNF10, and head-to-head splicing of circRNF10 was determined using Sanger sequencing (Figure 2A). To further verify the accuracy of the bioinformatics analysis, we analyzed circRNF10 expression in 30 pairs of BC and paracancerous tissues using qRT-PCR analysis. Our findings showed that circRNF10 was significantly reduced in BC tissues (Figure 2B), which was the same result obtained in bioinformatics analysis. We then examined circRNF10 expression in BC cells. BT-549 and MDA-MB-231 cells revealed consistently low expression of circRNF10 (Figure 2C).

To characterize circRNF10, two sets of primers were designed: divergent and convergent. These two sets of primers were used to amplify the circular form of circRNF10 and the linear form of RNF10. Complementary DNA (cDNA) and genomic DNA (gDNA) extracted from MDA-MB-231 and BT-549 cells were used as templates, and the results revealed that circRNF10 could only be amplified in cDNA, but RNF10 mRNA was amplified in both gDNA and cDNA (Figure 2D).

Furthermore, the stability and localization of circRNF10 were further assessed by actinomycin D, RNase R, and FISH assays. According to actinomycin D and RNase R assay results, circRNF10 mRNA was more stable than RNF10 mRNA after actinomycin D and RNase R treatment (Figure 2E,F). Subsequent FISH assays demonstrated that circRNF10 was primarily localized to the cytoplasm rather than the nucleus of MDA-MB-231 and BT-549 cells (Figure 2G). Altogether, the above results show that circRNF10 was reduced in BC tissue and localized predominantly to the cytoplasm of BC cells.

### 3.3. The Expression of circRNF10 Is Associated with BC Progression

**Figure 3:**
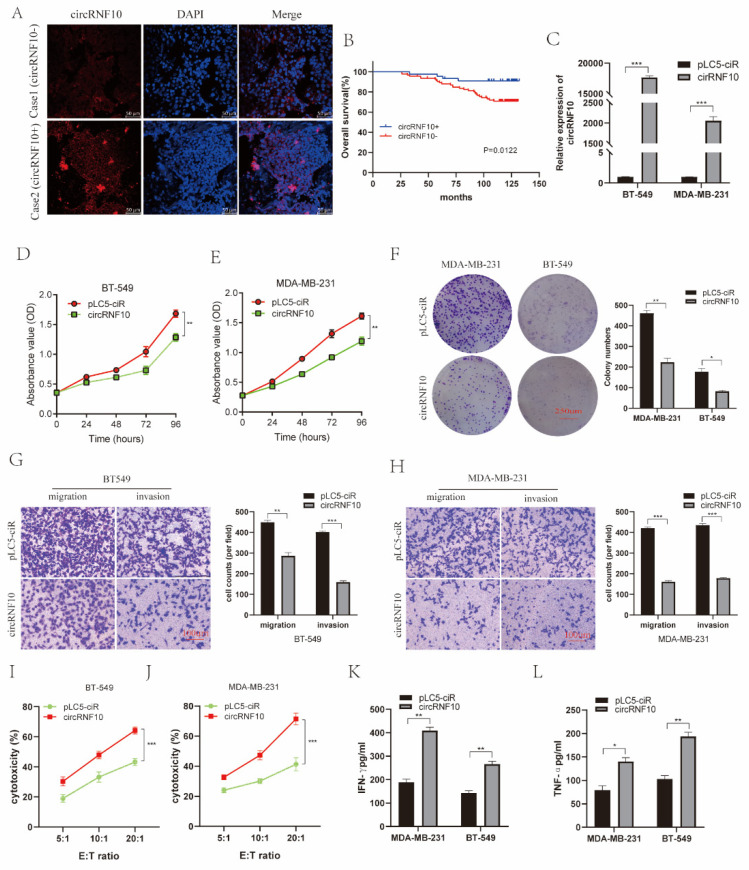
circRNF10 is related to BC progression, suppresses BC cell proliferation, migration, and invasion, and enhances the killing efficiency of NK-92MI cells against BC cells. (**A**) Level of circRNF10 in BC tissue specimens was observed by FISH analysis. Scale bar, 50 μm. (**B**) Survival analysis indicated that enhanced circRNF10 expression showed a favorable prognosis for BC patients. (**C**) Level of circRNF10 in BC cells after transfection with circRNF10 was observed using qRT-PCR. *** *p* < 0.001. (**D**,**E**) CCK8 assay indicated that overexpression of circRNF10 significantly suppressed proliferation of BT-549 and MDA-MB-231 cells, respectively. ** *p* < 0.01. (**F**) Colony formation assay indicated that the number of cell clones was markedly decreased after upregulation of circRNF10 expression in BC cells. Scale bar, 250 μm.* *p* < 0.05, ** *p* < 0.01. (**G**,**H**) Transwell assay indicated that overexpression of circRNF10 suppressed the migration and invasion abilities of BT-549 and MDA-MB-231 cells, respectively. Scale bar, 100 μm.** *p* < 0.01, *** *p* < 0.001. (**I**,**J**) Cytotoxicity of NK-92MI cells was detected using the LDH assay after co-culture with overexpression of circRNF10 in BT-549 (**I**) or MDA-MB-231 (J) cells with 5:1, 10:1 and 20:1 effect-to-target ratios. *** *p* < 0.001. (**K**,**L**) Protein levels of IFN-γ and TNF-α, respectively, in supernatant of NK-92MI cells with co-cultured overexpression of circRNF10 BC cells were measured by ELISA. * *p* < 0.05, ** *p* < 0.01. All of the experiments were repeated three times.

To further investigate the correlation between circRNF10 expression and BC prognosis, we detected the expression of circRNF10 in 140 BC tissues by FISH analysis with tissue microarrays (Figure 3A). The survival analysis results indicated that a higher level of circRNF10 was markedly related to a favorable prognosis (Figure 3B). This demonstrates that circRNF10 may have an anti-oncogenic role in BC progression.

### 3.4. circRNF10 Suppresses BC Cell Proliferation, Invasion, and Migration and Enhances the Killing Efficiency of NK-92MI Cells against BC Cells

To evaluate the biological function of circRNF10 in BC cells, BT-549 and MDA-MB-231 were transfected with a circRNF10 overexpression vector. qRT-PCR results revealed that circRNF10 expression was significantly enhanced (Figure 3C). CCK-8 and clone formation assays indicated that overexpressing circRNF10 suppressed the proliferation of BC cells (Figure 3D–F). The Transwell assay indicated that circRNF10 overexpression repressed the migration and invasion abilities of BC cells (Figure 3G,H). We then detected the cytotoxicity of NK cells toward BC cells by the LDH release assay. The cytotoxicity of NK-92MI cells was significantly increased in the circRNF10-overexpressing MDA-MB-231 cell group compared to the pLC5-ciR group (Figure 3I), and NK-92MI cells co-cultured with BT-549 cells also showed an increased ability to kill circRNF10-overexpressing BC cells (Figure 3J). We additionally observed whether overexpression of circRNF10 can promote the release of cytokines by NK-92MI cells. Secreted levels of TNF-α and IFN-γ in the supernatant of NK-92MI cells with co-cultured overexpression of circRNF10 breast cancer cells were detected by ELISA (Figure 3K,L), which indicated that overexpression of circRNF10 caused enhanced cytokine secretion by NK-92MI cells, thus potentiating their cytotoxic effect. The above results suggest that circRNF10 can inhibit BC cell proliferation, migration, and invasion and enhance the killing efficiency of NK-92MI cells against BC cells.

### 3.5. circRNF10 Acts as a Sponge for miR-934 in BC Cells

**Figure 4:**
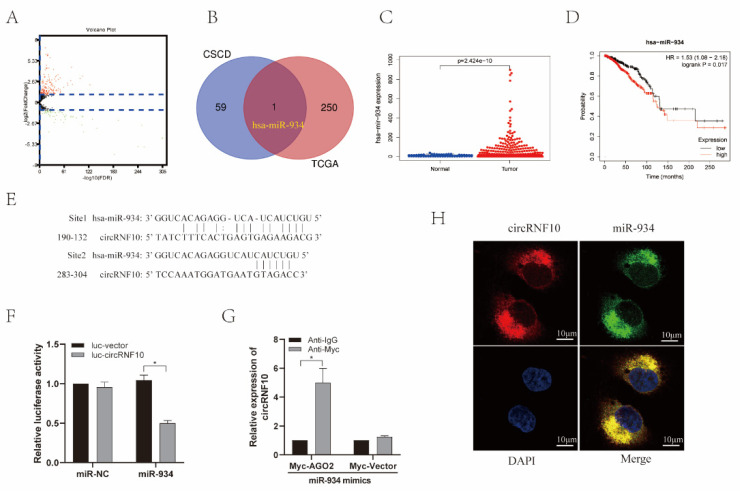
circRNF10 serves as a miR-934 sponge in BC cells. (**A**) Volcano plot of DEmiRNAs based on the TCGA database. (**B**) DEmiRNAs and predicted target miRNAs were determined by the intersection of the Venn diagram. (**C**) Levels of has-miR-934 were observed using the TCGA database. (**D**) Association between has-miR-934 expression and OS in BC patients was observed by the Kaplan–Meier Plotter database. (**E**) Predicted potential binding sites of circRNF10 and has-miR-934. (**F**) Luciferase promoter assay was performed to determine the luciferase activity of luc-circRNF10 in MDA-MB-231 cells co-transfected with miR-934 mimics. Experiments were repeated thrice. * *p* < 0.05. (**G**) RIP assay was performed to investigate the combination of circRNF10 and miR-934. Experiments were repeated thrice. * *p* < 0.05. (**H**) Colocalization of circRNF10 and has-miR-934 in MDA-MB-231 cells was determined by the FISH assay. Scale bar, 10 μm.

The CSCD database was used to predict circRNA-bound miRNAs, and 60 target miRNAs of circRNF10 were predicted. FDR < 0.05 and |log2FC| ≥ 1 were used as thresholds to analyze the differentially expressed miRNAs (DEmiRNAs) in BC tissues from the TCGA database. We screened 251 DEmiRNAs in BC, of which 64 were highly expressed and 187 were underexpressed (Figure 4A). Then, we determined the intersection of predicted target miRNAs with DEmiRNAs by constructing a Venn diagram and identified has-miR-934 as a major regulatory gene in BC (Figure 4B). We then investigated the expression of has-miR-934 in BC in the TCGA database. The results demonstrated that has-miR-934 expression was higher in BC tissues than in adjacent normal tissues (Figure 4C). Survival analysis showed that a higher level of has-miR-934 was markedly related to an adverse BC prognosis (Figure 4D). Furthermore, the bioinformatics prediction analysis demonstrated that there were two predicted binding sites between circRNF10 and miR-934 (Figure 4E). A luciferase promoter assay was performed to determine the effect of miR-934 on the activity of circRNF10, and the results indicated that miR-934 significantly suppressed the luciferase activity of luc-circRNF10 (Figure 4F). Then, a RIP assay was used to detect the interactions between circRNF10 and miR-934, and the results showed that circRNF10 was significantly increased using anti-Myc in circRNF10-overexpressing MDA-MB-231 cells by co-transfection of miR-934 mimics and Myc-AGO2 (Figure 4G). We further observed the subcellular colocalization of circRNF10 and miR-934 in MDA-MB-231 cells by FISH assay. The results indicated that circRNF10 and miR-934 were mainly present in the cytoplasm (Figure 4H). The above results indicate that circRNF10 may act as a sponge for miR-934 in BC cells.

### 3.6. miR-934 Enhances BC Cell Proliferation, Invasion, and Migration and Reduces the Killing Efficiency of NK-92MI Cells against BC Cells

**Figure 5:**
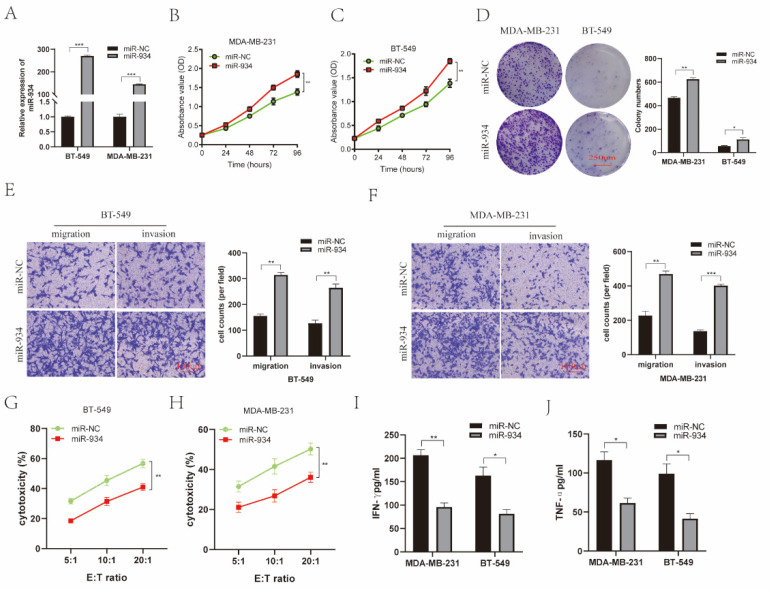
miR-934 promotes proliferation, migration, and invasion of BC cells and reduces the killing efficiency of NK-92MI cells against BC cells. (**A**) Expression of miR-934 in BC cells was observed by qRT-PCR. *** *p* < 0.001. (**B**,**C**) CCK8 assay revealed that miR-934 overexpression significantly enhanced proliferation of MDA-MB-231 and BT-549, respectively. ** *p* < 0.01. (**D**) Colony formation assay demonstrated that the number of cell clones was markedly increased after upregulation of miR-934 expression in MDA-MB-231 and BT-549 cells. Scale bar, 250 μm. * *p* < 0.05, ** *p* < 0.01. (**E**,**F**) Transwell experimental analysis indicated that overexpression of miR-934 promoted the migration and invasion abilities of MDA-MB-231 and BT-549 cells, respectively. Scale bar, 100 μm. ** *p* < 0.01, *** *p* < 0.001. (**G**,**H**) Cytotoxicity of NK-92MI cells was detected using the LDH assay after co-culture with overexpressing miR-934 in BT-549 (G) or MDA-MB-231 (**H**) cells with 5:1, 10:1, and 20:1 effect-to-target ratios. ** *p* < 0.01. (**I**,**J**) Levels of IFN-γ (**I**) and TNF-α (**J**) in supernatant of NK-92MI cells with co-cultured overexpressing miR-934 BC cells were observed by ELISA. * *p* < 0.05, and ** *p* < 0.01. All experiments were repeated thrice.

To further investigate the molecular functions of miR-934 in BC cells, we first constructed an miR-934 overexpression system by using miR-934 mimics in MDA-MB-231 and BT-549 cells (Figure 5A). Then, the proliferative ability of BC cells in different groups was determined by CCK-8 and colony formation assays. The results revealed that miR-934 overexpression enhanced the proliferative ability of BC cells (Figure 5B–D). Subsequently, the effect of miR-934 on invasion and migration of BC cells was detected by a Transwell assay. Our data demonstrate that miR-934 overexpression significantly increased the migration and invasion of BC cells (Figure 5E,F). The cytotoxicity of NK-92MI cells on BC cells was investigated by LDH assay, and NK-92MI cells co-cultured with BC cells showed a reduced ability to kill circRNF10-overexpressing BC cells (Figure 5G,H). An ELISA further showed that overexpression of miR-934 caused reduced IFN-γ and TNF-α secretion by NK-92MI cells (Figure 5I,J). Overall, our data demonstrate that miR-934 promotes proliferation, invasion, and migration but reduces the killing efficiency of NK-92MI cells against BC cells.

### 3.7. PTEN Was the Target Gene of miR-934

**Figure 6:**
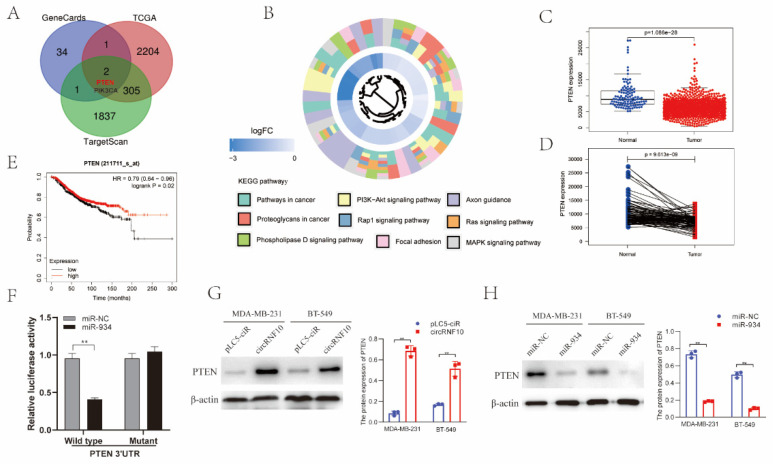
PTEN was the target gene of miR-934. (**A**) Potential targets of miR-934 were predicted based on TargetScan, GeneCards, and TCGA databases. (**B**) KEGG signaling pathway analysis of miR-934 target genes. (**C**) Expression of PTEN mRNA in 1109 BC specimens and 113 normal specimens was determined based on the TCGA database. (**D**) mRNA level of PTEN in 112 pairs of BC and normal tissues was determined based on the TCGA database. (**E**) Association between PTEN expression and OS in BC was determined by the Kaplan–Meier Plotter database. (**F**) Luciferase reporter activity of MDA-MB-231 cells co-transfected with luciferase reporters containing wild-type or mutant PTEN 3′UTR vector and miR-934 mimics or NC was detected by the dual-luciferase reporter assay. ** *p* < 0.01. (**G**) Protein levels of PTEN in circRNF10 overexpression systems were observed by Western blotting. ** *p* < 0.01. (**H**) Protein levels of PTEN transfected with miR-NC or miR-934 mimics were investigated by Western blotting. ** *p* < 0.01. The uncropped western blot figures for relevant results are shown in Figure S1.

Potential targets of miR-934 were predicted based on Targetscan. Furthermore, these potential targets of miR-934 were predicted based on TargetScan. These target genes were crossed with breast cancer-related genes in the GeneCards database and differential genes in the TCGA database, and two target genes were finally obtained: PTEN and PIK3CA (Figure 6A). We selected PTEN to perform subsequent validation. KEGG signaling pathway results showed that nine pathways were related to the target genes of miR-934 (Figure 6B). Among these pathways, the PI3K-Akt signaling pathway was most significantly associated with the target genes. We then investigated PTEN expression in BC. The results demonstrated that PTEN expression was lower in 1109 BC specimens than in 113 normal specimens (Figure 6C). Furthermore, we determined PTEN expression in 112 pairs of BC and normal specimens, and the results indicated that PTEN was significantly downregulated in BC (Figure 6D). The results of the survival analysis also revealed that PTEN expression was related to BC prognosis (Figure 6E). The dual-luciferase reporter assay further indicated that the miR-934 mimic greatly suppressed the luciferase reporter activity of the wild-type PTEN luciferase plasmid rather than the PTEN mutant plasmid (Figure 6F). The expression of the PTEN protein was also investigated in the circRNF10 overexpression system. We discovered that PTEN protein expression was significantly enhanced in the circRNF10 group (Figure 6G). Furthermore, we investigated the relationship between miR-934 and PTEN. As shown in Figure 6H, miR-934 inhibited the protein expression of PTEN in MDA-MB-231 and BT-549 cells. Altogether, our research data show that PTEN is the target gene of miR-934, and PTEN expression can be influenced by circRNF10.

### 3.8. circRNF10 Suppresses BC Cell Proliferation, Migration, and Invasion and Enhances the Killing Efficiency of NK-92MI Cells on BC Cells via miR-934

**Figure 7:**
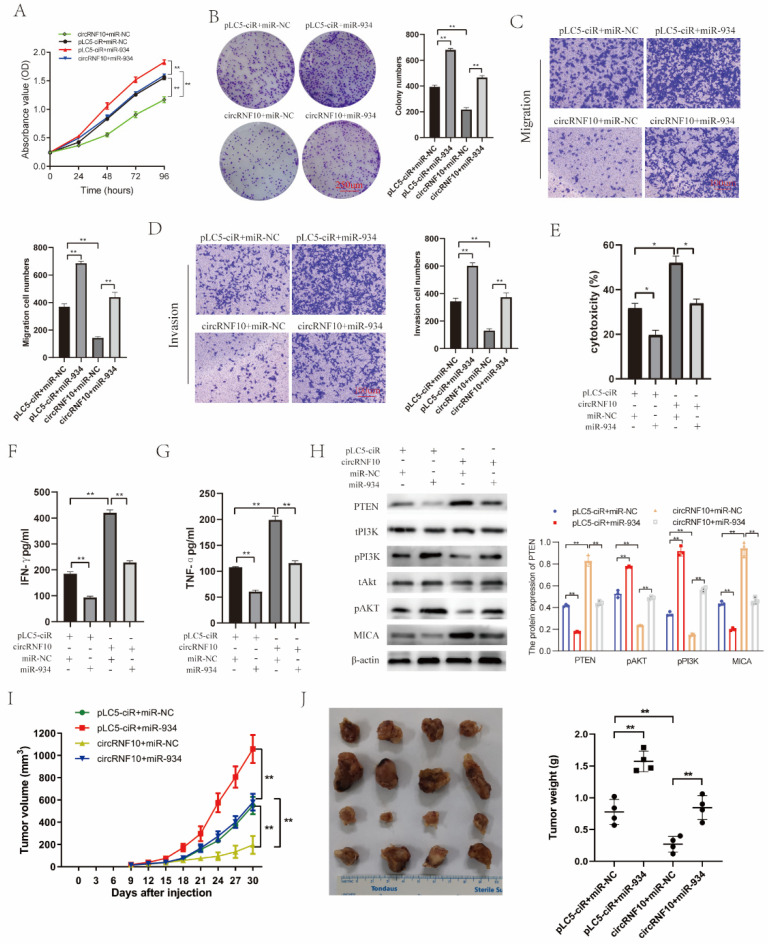
circRNF10 regulates miR-934 to affect BC cell proliferation, migration, and invasion, as well as the killing efficiency of NK-92MI cells on BC cells and may have an effect on the PI3K-Akt pathway. BC cells were co-transfected with circRNF10, pLC5-ciR, miR-934, or NC. (**A**,**B**) Proliferation of BC cells was detected by CCK-8 and colony formation assays, respectively. Scale bar, 250 μm. Experiments were repeated thrice. ** *p* < 0.01. (**C**,**D**) Cell migration and invasion were determined using Transwell migration and invasion experiments, respectively.Scale bar, 100 μm. Experiments were repeated thrice. ** *p* < 0.01. (**E**) NK-92MI cell killing ability against MDA-MB-231 cells was determined by the LDH assay. Experiments were repeated thrice. * *p* < 0.05. (**F**,**G**) Protein levels of IFN-γ (**F**) and TNF-α (**G**) in supernatant of NK-92MI cells were observed by ELISA. Experiments were repeated thrice. ** *p* < 0.01. (**H**) Protein levels of PTEN, tPI3k, pPI3k, tAKT, pAKT, MICA, and β-catenin were detected by Western blotting. ** *p* < 0.01. (**I**,**J**) Tumor growth and tumor weight were evaluated. ** *p* < 0.01. The uncropped western blot figures for relevant results are shown in Figure S1.

To investigate how circRNF10 performs its biological function in BC cells, we used circRNF10, miR-934, or the combination of both to transfect MDA-MB-231 cells, then determined the cell proliferation, invasion, and migration of BC cells and the killing efficiency of NK-92MI cells on BC cells. CCK-8 and colony formation ability assays (Figure 7A,B) revealed that miR-934 overexpression alone notably enhanced the proliferation ability of MDA-MB-231 cell lines, but the increased effect was restored when circRNF10 was plugged at the same time. Similarly, Transwell assay results demonstrated that miR-934 overexpression could attenuate the inhibitory role of circRNF10 overexpression in migration and invasion of MDA-MB-231 cells (Figure 7C,D). In addition, miR-934 mimics abrogated the circRNF10-induced improvement of NK-92MI cell killing ability against MDA-MB-231 cells, as evidenced by impaired NK-92MI cell cytotoxicity in circRNF10-transfected cells following the introduction of miR-934 mimics (Figure 7E). Similarly, ELISA revealed that IFN-γ and TNF-α secretion was greatly enhanced by overexpression of circRNF10, while miR-934 compromised this effect (Figure 7F,G). To further verify whether circRNF10 acts as an anti-oncogene via the PI3K-Akt pathway by sponging miR-934, we attempted to explore the protein expression level of the PI3K-Akt pathway in MDA-MB-231 cells. We discovered that the expression of PTEN and MICA was significantly upregulated in the circRNF10 overexpression group, while these two proteins showed downregulated expression after transfection with miR-934 mimics (Figure 7H). However, the protein expression of pPI3K and pAkt had the reverse tendency (Figure 7H). Moreover, by constructing mouse xenografts, we found that circRNF10 overexpression inhibited the growth of xenograft tumor, but the inhibiting effect was abrogated when miR-934 was plugged (Figure 7I,J). Overall, these findings reveal that circRNF10 regulates miR-934 to influence BC cell proliferation, migration, invasion, and enhance the killing efficiency of NK-92MI cells on BC cells, and could have an effect on the PI3K-Akt pathway.

## 4. Discussion

In the past few years, through the analysis of sequencing data, numerous circRNAs have been found in various types of diseases [18]. Numerous studies have discovered that circRNAs are abnormally expressed in various carcinomas and play crucial roles in carcinoma biology. Due to the resistance of circRNAs to exonucleases relative to the linear forms of mRNA, they are more advantageous as biomarkers for judging patient prognosis [19]. In addition, many circRNAs, such as circACTN4 [20], circIPO7 [21], circWWC3 [22], circREEP3 [23], and circCCDC134 [24], have been found to play major roles in the initiation and progression, chemoresistance, immune escape, and distant metastasis of tumors. Thus, studying the molecular mechanism of circRNAs in BC progression will provide a novel direction for clarifying the pathogenesis and refining the clinical treatment of BC.

In this study, circRNA microarray data were obtained from the GEO database, and the limma package was used to identify DECs. Among them, we discovered that circRNF10 was notably reduced in BC tissues and was distinctly related to an adverse BC prognosis. Enforced circRNF10 expression significantly suppressed BC cell proliferation, invasion, and migration abilities and enhanced the killing efficiency of NK-92MI cells toward BC cells. According to this biological phenomenon, we then investigated the possible role and mechanism of circRNF10 in BC cells. Studies have suggested that circRNAs have multiple crucial biological functions by acting as scaffolds for the assembly of protein complexes, regulating RNA splicing and gene transcription, and serving as microRNA sponges [11,25,26]. For exon-derived circRNAs, their role as microRNA sponges is presently receiving the most research attention. Given that circRNF10 is derived from exons five and six of the host RNF10 gene and localized predominantly in the cytoplasm, we reasoned that circRNF10 might function as an miRNA sponge. To confirm whether this was the case, we used the CSCD database to predict circRNA-bound miRNAs and bioinformatics prediction tools to predict circRNF10-bound miRNAs and verified the results with many experimental studies, including FISH, the luciferase reporter assay, RIP, and Western blots. Fortunately, our findings are consistent with the supposition that circRNF10 functions as a sponge for miR-934. In addition, we screened the predicted target gene, PTEN, of miR-934 using bioinformatics methods and further verified that the tumor suppressor gene PTEN was indeed the target gene of miR-934. Western blot results indicate that PTEN expression can be influenced not only by miR-934 but also by circRNF10. The rescue experiment data indicate that the effect of suppressing the proliferation, migration, and invasion of BC cells and enhancing the killing efficiency of NK-92MI cells against BC cells caused by overexpression of circRNF10 was offset by miR-934 mimics. In addition, our data indicate that circRNF10 functions as a sponge for miR-934 to influence PTEN expression and PI3k/Akt signaling in BC.

Studies have shown that miR-934 can induce M2 polarization of macrophages to stimulate colorectal cancer metastasis [27]. Furthermore, miR-934 has been demonstrated to act as an oncogene in pancreatic carcinoma, lung adenocarcinoma, and bladder cancer [28,29]. However, the correlation between miR-934 and BC has not been discussed. Our research found that miR-934 can enhance the proliferation, invasion, and migration of BC cells and reduce the killing efficiency of NK-92MI cells against BC cells by targeting the 3′UTR of the target gene PTEN. In addition, overexpression of circRNF10 may allow it to function as a sponge for miR-934 and reverse miR-934-mediated biological functions.

Within the tumor microenvironment, NK cells play an essential role in host defense against virus infection and tumor progression [30]. Recently, emerging evidence has shown that circular RNAs can affect tumor progression by affecting the killing activity of NK cells. For instance, the susceptibility of hepatocellular carcinoma cells to NK cells was found to be significantly influenced by the expression of hsa_circ_0007456 [31]. circUHRF1 can suppress NK cell biological function by enhancing the level of TIM-3 by sponging miR-449c-5p in hepatocellular carcinoma [32]. In bladder cancer, circRHOT1 can induce SMAD5 expression to regulate tumorigenic properties and NK cell-mediated toxicity by targeting miR-3666 [33]. In our research, we found that circRNF10 enhanced the expression of MICA, the receptor of NKG2D, to regulate NK cell-mediated toxicity by suppressing PI3K/Akt signaling in breast cancer. Based on our results, circRNF10 is expected to be a target for further investigation of the function of NK cells in BC.

**Figure 8:**
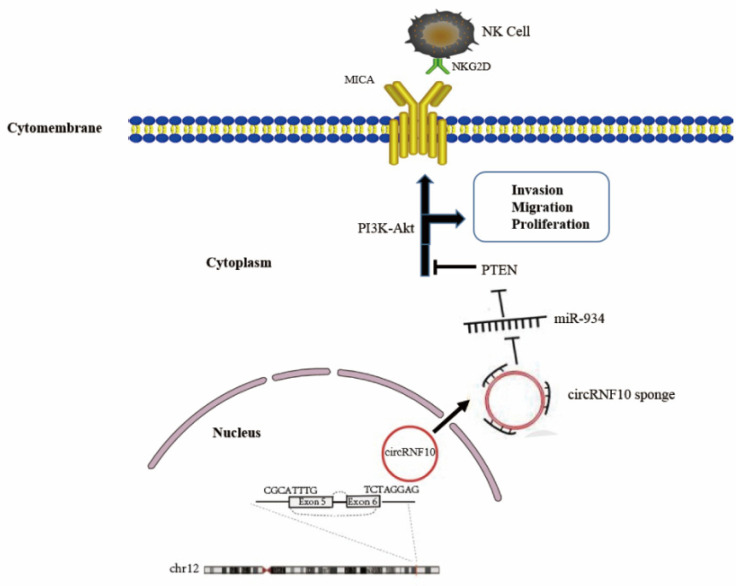
Possible mechanism by which circRNF10 suppress proliferation, invasion, and migration of BC cells and enhances killing efficiency of NK-92MI cells against BC cells by acting as an miR-934 sponge to regulate PTEN expression and PI3k/Akt/MICA signaling in BC cells.

This study found that circRNF10 was notably reduced and suppressed the proliferation, migration, and invasion of BC cells and enhanced the killing efficiency of NK-92MI cells against BC cells. Mechanically, circRNF10 serves as a sponge for miR-934 to further regulate PTEN expression and PI3k/Akt signaling in breast cancer (Figure 8). These results prove that circRNF10 exerts a biological function through the miR-934-PTEN-PI3k/Akt signaling pathway axis, which expands our knowledge about the molecular mechanism of BC initiation and development.

## 5. Conclusions

In conclusion, our study results demonstrate that circRNF10 is downregulated in BC tissues and related to the prognosis of patients with BC. Furthermore, it can function as a regulator in BC cell proliferation, migration, and invasion and affect the killing efficiency of NK-92MI cells on BC cells. Mechanistically, circRNF10 serves as a sponge for miR-934 to affect PTEN expression and inhibit the PI3k/Akt/MICA signaling pathway. Our study provides novel insights into the pathogenetic mechanisms of BC and indicates that circRNF10 has the potential to be a novel target for molecular therapy of BC.

## Figures and Tables

**Table 1:** Basic characteristics of seven differently expressed circRNAs.

circRNA	Genomic Length	Position	Strand	Gene Symbol	Best Transcript	Regulation
hsa_circ_0028899	401	chr12:120995084-120995485	+	RNF10	NM_014868	down
hsa_circ_0000376	48,782	chr12:11199618-11248400	-	PRH1-PRR4	NR_037918	down
hsa_circ_0000519	98	chr14:20811436-20811534	-	RPPH1	NR_002312	up
hsa_circ_0000375	401	chr12:6657590-6657991	-	IFFO1	NM_080730	down
hsa_circ_0000517	88	chr14:20811404-20811492	-	RPPH1	NR_002312	up
hsa_circ_0003645	7205	chr16:19656207-19663412	+	C16orf62	NM_020314	up
hsa_circ_0000516	85	chr14:20811398-20811483	+	RPPH1	NR_002312	up

## Data Availability

All data in this study are available from the corresponding author upon request.

## Supplementary Materials

The following supporting information can be downloaded at: https://www.mdpi.com/article/10.3390/cancers14235862/s1, Figure S1: The uncropped western blot figures for relevant results. Table S1: The primers used in the PCR amplification.
