# Supplementary material for: circRNF10 Regulates Tumorigenic Properties and Natural Killer Cell-Mediated Cytotoxicity against Breast Cancer through the miR-934/PTEN/PI3k-Akt Axis
Source: Cancers (Basel). 2022 Nov 28;14(23):5862. doi: 10.3390/cancers14235862 (PMC9739140; doi:10.3390/cancers14235862)
Supplement: Supplementary file 1 [file cancers-14-05862-s001.zip › Figure S1.pdf]

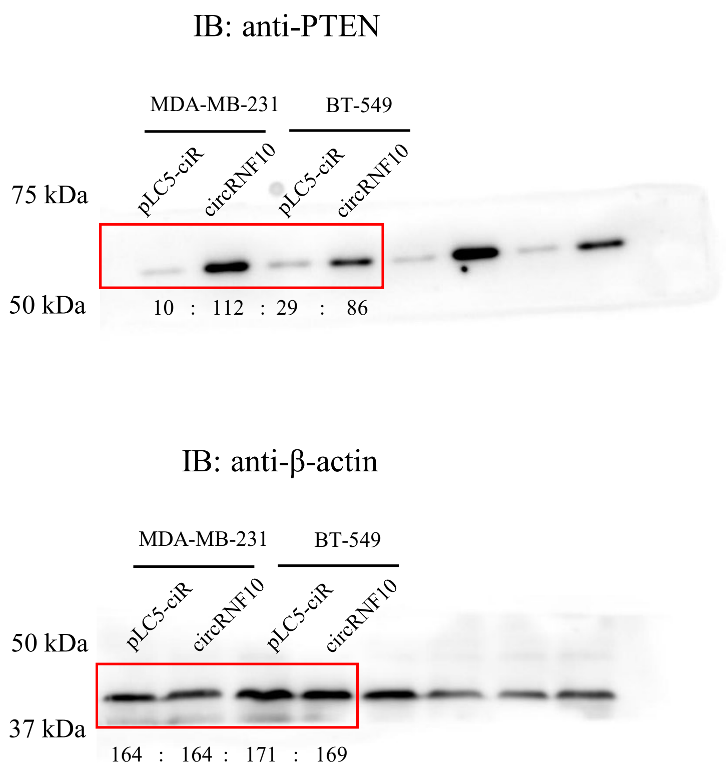

Uncropped western blot of Figure 6G.

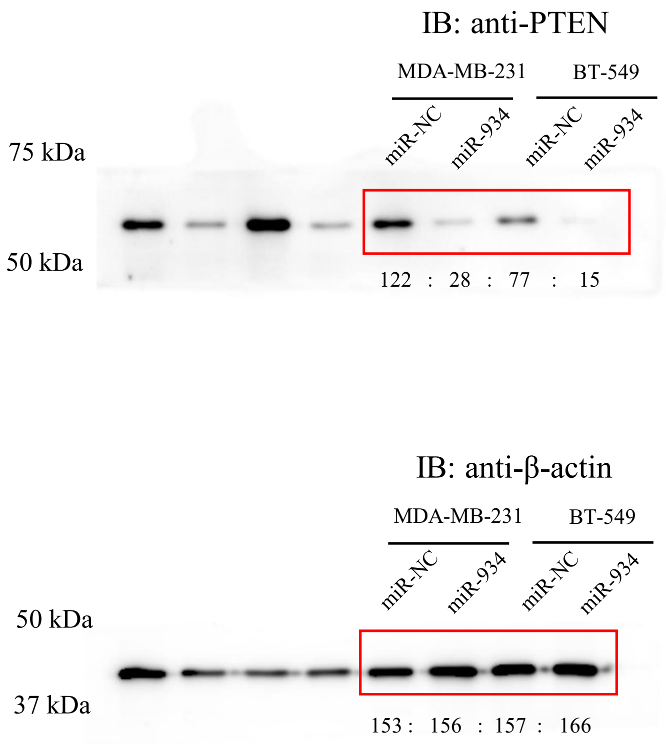

Uncropped western blot of Figure 6H.

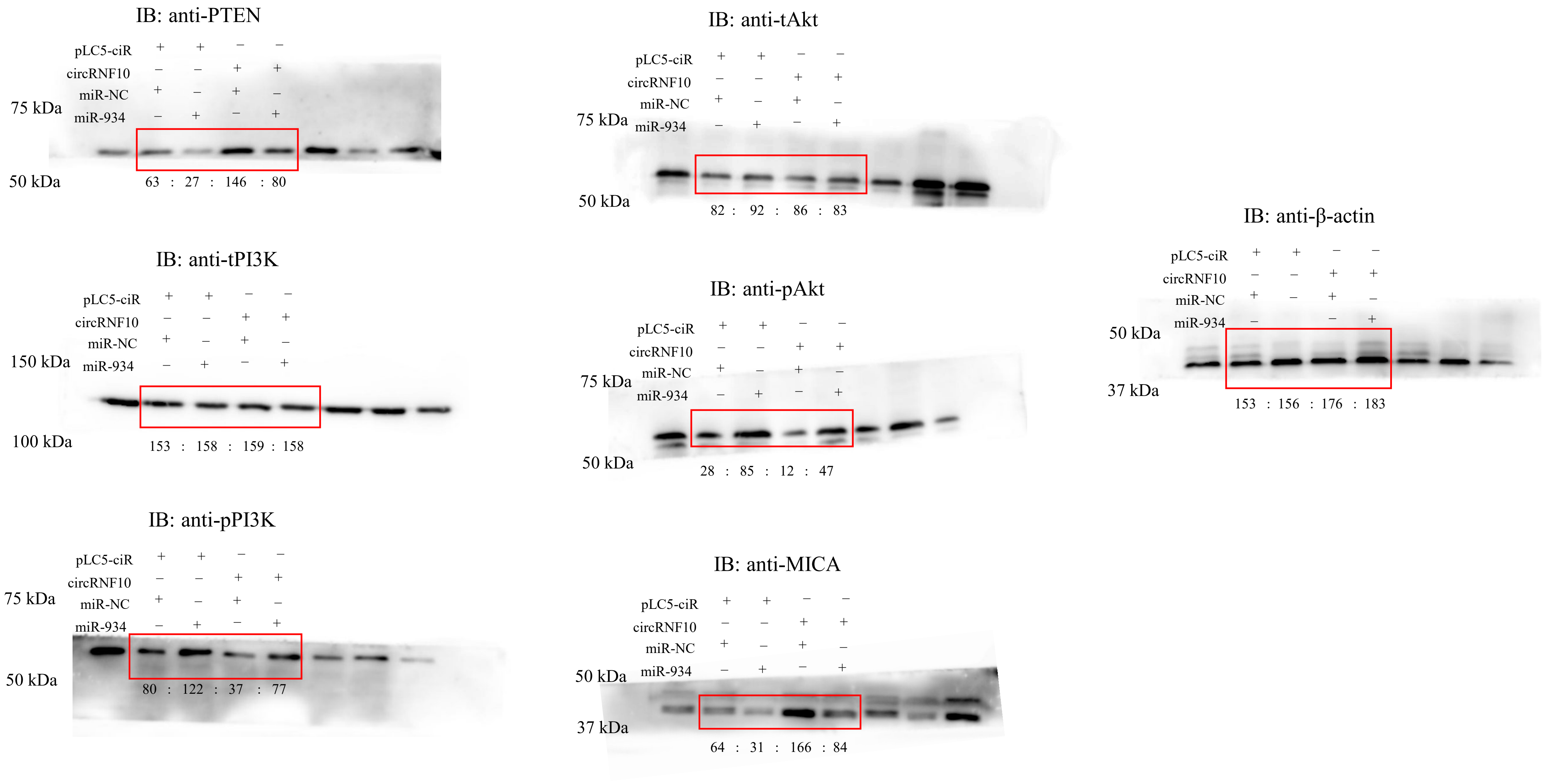

Figure S1. Uncropped western blot of Figure 7H.
