# Supplementary material for: circRNF10 Regulates Tumorigenic Properties and Natural Killer Cell-Mediated Cytotoxicity against Breast Cancer through the miR-934/PTEN/PI3k-Akt Axis
Source: Cancers (Basel). 2022 Nov 28;14(23):5862. doi: 10.3390/cancers14235862 (PMC9739140; doi:10.3390/cancers14235862)
Supplement: Supplementary file 1 [file cancers-14-05862-s001.zip › Table S1.pdf]

Supplementary Table S1 The primers used in the PCR amplification

Genes	Primers
**circRNF10**	Forward, 5’-CCATTCATCTAGGAGCGCATT-3’
	Reverse, 5’-GCAGTAGGTGGATAGAGGCATATT-3’
**RNF10**	Forward, 5’- ACCCAAGAGCGATGGAAAGA-3’
	Reverse, 5’- AACTGGGCAGGGCTAAACTC-3’
**GAPDH**	Forward, 5’-CGCTGAGTACGTCGTGGAGTC-3’
	Reverse, 5’-GCTGATGATCTTGAGGCTGTTGTC-3’
**U6**	RT, 5’-AACGCTTCACGAATTTGCGT-3’
	Foward, 5’-CTCGCTTCGGCAGCACA-3’
	Reverse, 5’-AACGCTTCACGAATTTGCGT-3’
